# Offset analgesia and onset hyperalgesia with different stimulus ranges

**DOI:** 10.1097/PR9.0000000000000914

**Published:** 2021-03-24

**Authors:** Jens Fust, Maria Lalouni, Viktor Vadenmark Lundqvist, Emil Wärnberg, Karin B. Jensen

**Affiliations:** a Department of Clinical Neuroscience, Karolinska Institutet, Stockholm, Sweden; b Department of Neuroscience, Karolinska Institutet, Stockholm, Sweden

**Keywords:** Offset analgesia, Onset hyperalgesia, Thermal pain stimulation, Endogenous pain modulation

## Abstract

**Introduction:**

Offset analgesia (OA), a large reduction in pain after a brief increase in intensity of an otherwise stable painful stimulus, has been established by a large body of research. But the opposite effect, onset hyperalgesia (OH), a disproportional hyperalgesic response after a briefly decreased intensity of a painful stimulus, has only been investigated in one previous study.

**Objectives:**

The aim of this study was to induce OA and OH in healthy participants and explore the effects of different stimulus ranges (increase/decrease of temperature) on OA and OH.

**Methods:**

A total of 62 participants were tested in 2 identical experiments. Offset analgesia and OH conditions included 2 different temperature deviations (±1°C/±2°C) from initial temperature and were compared with a constant temperature (control).

**Results:**

Offset analgesia was successfully elicited in OA_1°C_ in experiment 1, and in OA_1°C_ and OA_2°C_ in experiment 2. Results indicate a continuous stimulus–response relationship between the stimulus range and the resulting hypoalgesic response. Onset hyperalgesia was only elicited in OH_2°C_ in experiment 1. Exploratory analysis showed that the lack of OH response in experiment 2 could be explained by sex differences, and that OA and OH responses were only weakly correlated.

**Conclusions:**

The asymmetry between pain responses after a brief temperature increase and decrease suggests that different mechanisms are involved in the pain responses to increasing and decreasing temperature. This asymmetry may also be explained by high temperatures in OA condition (+1°C/+2°C above baseline) that could be seen as salient “learning signals,” which augment the response to following changes in temperature.

## Abstract 2

A comparison between the effects of offset analgesia and onset hyperalgesia and how these effects relate to the stimulus range of thermal stimulation.

## 1. Introduction

The ability to feel pain is essential for our survival as it provides a warning signal for potential tissue damage.^10^ Yet, the perception of pain varies as a result of contextual adaptation.^4^ For example, pain intensity can be both upregulated and downregulated based on the temporal characteristics of a noxious stimulus.^5,9^ Evidence suggest that a noxious stimulus with fast onset is experienced as more painful than a stimulus with slow onset.^20^ In terms of the motivational aspects of pain, increasing painful stimulation may represent a strong signal of imminent tissue damage, and the opposite, decreasing painful stimulation, can be seen as a safety signal.^5^ The offset analgesia (OA) paradigm is an example of a manipulation of the temporal signature of noxious stimulation associated with hypoalgesia (we prefer the term hypoalgesia to analgesia, because pain is usually reduced, not eliminated).^7^ More specifically, the OA response represents a disproportional reduction of pain after a brief increase and decrease of an otherwise stable painful stimulus.

The function of the OA response is not entirely clear, even if it has been described in terms of a temporal contrast enhancement mechanism that amplifies changes in the afferent signal.^13,22^ The OA response is also associated with biological correlates related to central pain modulation, such as neural activation at the spinal level and the periaqueductal gray.^17,21^ In contrast to other experimental paradigms that measure central pain modulation, such as temporal summation and conditioned pain modulation, the OA response has not been suppressed by pharmacological blocking of specific neurotransmitter receptors.^8,11^ Patients with neuropathic and nociplastic pain syndromes often exhibit reduced OA responses compared with healthy populations, which suggest that the OA paradigm is sensitive to disrupted pain modulation associated with long-term pain.^12,14,16,18^

Offset analgesia demonstrates that fluctuating heat can produce *hypo*algesia, but less effort has been put on examining the opposite response, *hyper*algesia. Using a simple 2-step sequence of temperatures on the skin (48°C–49°C and 49°C–48°C), compared with constant heat, Mørch et al.^9^ demonstrated a disproportional decrease and increase of pain, respectively. In a recent study, Alter et al. produced “onset hyperalgesia” (OH) by inverting the standard OA 3-step sequence.^1^ Although the evidence is sparse, these studies indicate the existence of a bidirectional temporal contrast mechanism that can both amplify and weaken the pain response to dynamic noxious stimulation.

The aim of experiment 1 was to elicit hyperalgesia with an OH paradigm and compare with the pain response elicited by the standard OA paradigm. To further study the dynamic relationship between heat and pain, we also included 2 different stimulus ranges (ie, increase/decrease in temperature) for both the OA and OH sequence, resulting in 4 experimental conditions (OA_1°C_, OA_2°C_, OH_1°C_, and OH_2°C_), as well as a control condition with constant temperature. We hypothesized that hyperalgesic response could be produced using the OH paradigm, as well as hypoalgesic responses using the standard OA paradigm. Moreover, we hypothesized that a larger temperature range would produce larger hyperalgesic and hypoalgesic responses. Experiment 2 was a direct replication of experiment 1. The procedure was identical to experiment 1, but with a larger sample size to increase statistical power. Also, both men and women were tested (only women were included in experiment 1). Because the method of statistical analysis was decided post-hoc in experiment 1, experiment 2 was conducted to corroborate the results of the first experiment.

## 2. Methods

### 2.1. Participants

A total of 63 healthy participants were recruited for the study. One participant was excluded from experiment 2 before statistical analyses because the participant did not look at the numerical rating scale (NRS) scale during the pain stimulation. Twenty-one women (mean age: 24, SD = 2.7) were included in experiment 1 and 41 participants (22 women; mean age: 25, SD = 4.5) in experiment 2. Inclusion criteria required that participants were as follows: (1) women (in experiment 1, but not experiment 2), (2) aged 18–35 years, and (3) in good health. All participants were recruited through advertisement on university campuses in Stockholm and on the Internet. The regional Ethics Review Board in Stockholm approved the study (Dnr: 2018/1367-31/1), and all subjects gave written informed consent.

### 2.2. Procedure

**Figure 1.**
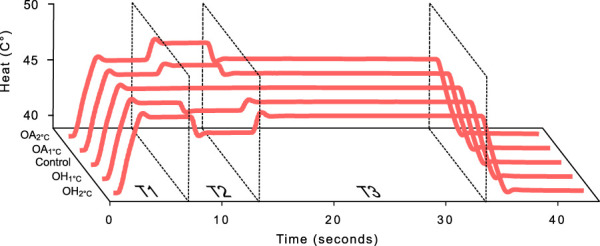
Mean heat stimulation (°C) during the 4 OA/OH conditions and the control condition. T1, T2, and T3 refer to 3 time intervals and denote T1 = T1 temperature premanipulation, T2 = temperature manipulation (±1°C or ±2°C), T3 = T1 temperature postmanipulation. OA, offset analgesia; OH, onset hyperalgesia.

Experiment 1 was conducted by J. Fust and M. Lalouni between January and February, 2019. Experiment 2 was conducted by V. Vadenmark Lundqvist between June and July, 2019. Both experiments followed the same procedure. Heat stimuli were administered with a thermal stimulator (Somedic Senselab AB, Hörby, Sverige). Temperature increased and decreased at a rate of 5°C/s. A 30 × 30 mm thermal probe was attached to participants' left calf. This site of the body was chosen because we wanted to design a procedure that could be implemented together with magnetic resonance imaging in future studies. Participants used a trackball to continuously rate their pain intensity on a NRS (without anchor words) that was displayed on a screen, marked with all integers ranging from 0 to 10 on a horizontal line. Participants were instructed verbally that 0 represented “no pain” and 10 “worst imaginable pain.” Numerical rating scale has been extensively used in pain research and is believed to be a valid measure of pain intensity in healthy populations.^6^ Individual pain sensitivity was calibrated before the experiment. We used individually calibrated temperature in the OA and OH paradigms, instead of fixed stimulus intensities, because we were primarily interested in pain intensity, not stimulus intensity per se. During the pain calibration, participants were exposed to 5 seconds heat stimuli ranging from 38°C to 50°C, with a 35 seconds break between each stimulus. After the calibration, participants were given an additional 15 seconds heat stimulation, set to each individual's 5 NRS, predicted from the calibration data. If the maximum pain rating ranged between 4 and 6 NRS, this temperature was used as the initial temperature in the experimental phase (from now on referred to as T1 temperature), otherwise the procedure was repeated with a higher or lower temperature until the desired pain rating was reached. The mean T1 temperature for experiment 1 and 2 was 47.1 (SD = 1.4°C). The OA and OH protocol can be divided up in 3 time intervals: T1, T2, and T3 (see Fig. 1 for a visual representation). During T1 (0–6.5 seconds), the temperature of the thermal stimulator increased from nonpainful temperature (38°C) to the individual calibrated T1 temperature. T1 continued approximately 5 seconds after the thermal stimulator reached the T1 temperature. During T2 (6.5–12 seconds), temperature either kept stable (control), increased 1°C or 2°C (OA_1°C_, OA_2°C_), or decreased 1°C or 2°C (OH_1°C_, OH_2°C_) from the T1 temperature. T2 continued for 5 seconds after the thermal stimulator reached assigned temperature. During T3 (12–33 seconds), the thermal stimulator returned to the T1 temperature, and approximately 20 seconds, the thermal stimulator returned to the nonpainful baseline temperature. Pain ratings were continuously registered until approximately 10 seconds after thermal stimulator returned to baseline temperature. The presentation order of the conditions was randomized for each participant. Participants were exposed to the 5 conditions once, and every condition was followed by a 50 seconds break with a baseline temperature of 38°C. The thermode was not moved between the conditions. All participants were able to tolerate the heat stimulations. We decided to use a slightly modified version of the OH design used by Alter et al.^1^ In addition to using 2 different temperature ranges, we also decided to keep the individually calibrated T1 temperature constant in all conditions, only varying the temperature during the second phase of the procedure, making comparisons between conditions easier. In the study by Alter et al.,^1^ the OH protocol was inverted so that the T2 temperature was assigned to T1 and T3, and the temperature decreased 1°C during T2.

### 2.3. Statistical analysis

The same statistical analyses were used for both experiments. First, we calculated mean pain ratings for each condition during the last 13 seconds of stimulation (from the time when the ratings started to diverge in T3 until temperature started to return to baseline). This time window was determined by visual inspection of the plotted data in experiment 1. Second, we performed 2 repeated measures 1 × 3 analysis of variance (ANOVA) on these mean pain ratings; one including the offset conditions and the control condition (OA_1°C_, OA_2°C_, and control) and one including the onset conditions and control condition (OH_1°C_, OH_2°C_, and control). Third, if the ANOVA models reached statistical significance, we performed paired *t*-tests to compare the pain ratings between individual conditions. Corrections for multiple comparisons were performed using the Benjamini–Hochberg method.^2^ A hypoalgesic response was determined as a difference between OA and the control condition, and a hyperalgesic response as a difference between OH and the control condition. Last, we conducted 2 exploratory analyses: with the purpose of (1) examining the level of symmetry between OA and OH responses; and (2) examining the effect of sex on OA and OH in experiment 2. To examine the level of symmetry between OA and OH responses, we analyzed the data with a similar method used in the study by Alter et al.^1^ Data from experiment 1 and 2 were merged. To be able to compare OA and OH, we subtracted the control conditions from each experimental condition and inverted the OA conditions. Then, we computed subtracted offset effects and onset effects for each participant and stimulus range. The subtracted offset effect and onset effects were defined as the difference between the minimum rating during T2 (9–20 seconds) and maximum rating during T3 (20–33 seconds). Because of the delayed perception to heat stimulation, we decided to prolong the window for analysis for T2. Finally, we calculated Pearson correlation coefficients between the offset effect and the onset effect for each stimulus range. To explore if there were any sex-related differences in OA and OH in experiment 2, we subtracted mean pain ratings of the control condition from the mean pain ratings of the offset and onset conditions during the last 13 seconds of stimulation, and performed 2-sample *t* test for each condition, comparing men and women. One participant was excluded from these analyses because the participant did not want to report their sex. All calculations were made using Python 3.7.5. Repeated measures ANOVA was calculated using AnovaRM from the Python library Statsmodels 0.10.1.^15^ Analysis scripts are available on Open Science Framework (https://osf.io/uh678/).

## 3. Results

**Figure 2.**
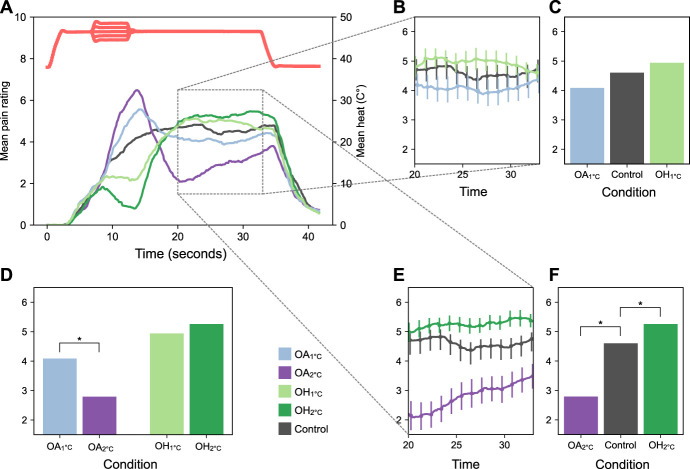
Results from experiment 1. (A) Mean pain ratings (left axis) and mean temperature (right axis) during OA_1°C_, OA_2°C_, OH_1°C_, OH_2°C_, and control condition. (B) Mean pain ratings for ±1°C conditions and control during the last 13 seconds of heat stimulation (error bars: ±1 within-subject standard error of the mean). (C) Comparison between pain ratings for ±1°C conditions and control during the last 13 seconds of heat stimulation. (D) Comparisons between pain ratings between ±1°C and +2°C conditions during the last 13 seconds of heat stimulation. (E) Mean pain ratings for ±2 conditions and control during the last 13 seconds of heat stimulation (error bars: ±1 within-subject standard error of the mean). (F) Total mean pain ratings for ±1 conditions and control condition during the last 13 seconds of heat stimulation. OA, offset analgesia; OH, onset hyperalgesia.

In experiment 1 (Fig. 2), we found a significant main effect of condition on pain ratings in the OA model, F(2, 40) = 10.86, *P* < 0.001. Post-hoc tests showed that there was a significant hypoalgesic response during OA_2°C_, *t*(20) = 4.57, *P* < 0.001 but not during OA_1°C_. The hypoalgesic response was stronger in OA_2°_ compared with OA_1°C,_ *t*(20) = 3.15, *P* = 0.008. We also found a significant main effect of condition on pain ratings in the OH model, F(2, 40) = 3.72, *P* = 0.033. Post-hoc tests revealed a significant hyperalgesic response during OH_2°C_, *t*(20) = −3.19, *P* = 0.007, but not during OH_1°C_, and no significant difference between OH_2°C_ and OH_1°C_.

**Figure 3.**
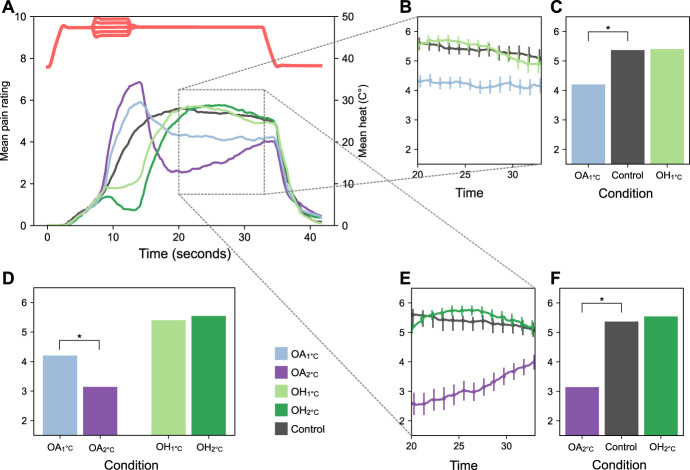
Results from experiment 2. (A) Mean pain ratings (left axis) and mean temperature (right axis) during OA_1°C_, OA_2°C_, OH_1°C_, OH_2°C_, and control condition. (B) Mean pain ratings for ±1°C conditions and control during the last 13 seconds of heat stimulation (error bars: ±1 within-subject standard error of the mean). (C) Comparison between pain ratings for ±1°C conditions and control during the last 13 seconds of heat stimulation. (D) Comparisons between pain ratings between ±1°C and +2°C conditions during the last 13 seconds of heat stimulation. (E) Mean pain ratings for ±2 conditions and control during the last 13 seconds of heat stimulation (error bars: ±1 within-subject standard error of the mean). (F) Total mean pain ratings for ±1 conditions and control condition during the last 13 seconds of heat stimulation. OA, offset analgesia; OH, onset hyperalgesia.

In experiment 2 (Fig. 3), we found a significant main effect of condition on pain ratings in the OA model, F(2, 80) = 39.10, *P* < 0.001. Post-hoc tests showed that there was a significant hypoalgesic effect during both OA_1°C_, *t*(40) = 5.39, *P* < 0.001 and OA_2°C_, *t*(40) = 6.97, *P* < 0.001. As in the first experiment, the hypoalgesic effect was stronger during OA_2°_ than OA_1°C,_ *t*(40) = 4.51, *P* < 0.001. The main effect of condition on pain ratings in the OH model was not significant, F(2, 80) = 3.72, *P* = 0.050. In the exploratory analysis, we found that women had significantly higher pain rating scores compared with men in OH_2°C_, *t*(39) = 2.16, *P* = 0.038, but no significant sex differences in the other conditions.

**Figure 4.**
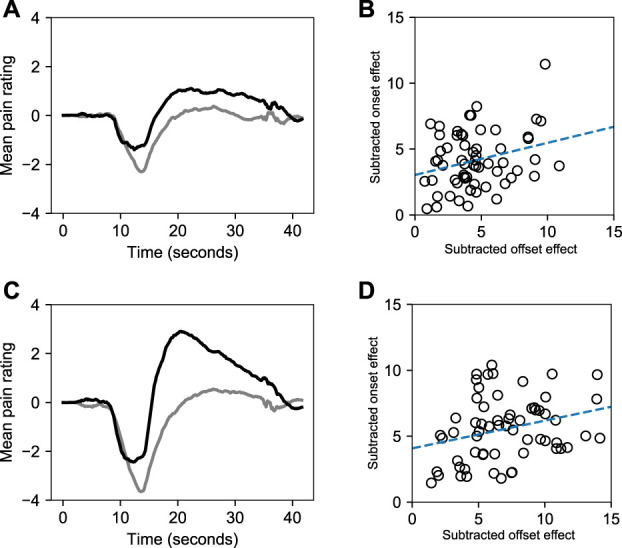
Results from exploratory analysis of OA OH symmetry. (A) The gray line represents the difference between pain ratings during OH_1°C_ and control. The black line represents the inverted difference between pain ratings during OA_1°C_ and control. (B) Correlation between subtracted offset effect and subtracted onset effect during ±1°C conditions. (C) The gray line represents the difference between pain ratings during OH_2°C_ and control. The black line represents the inverted difference between pain ratings during OA_2°C_ and control. (D) Correlation between subtracted offset effect and subtracted onset effect during ±2°C conditions. OA, offset analgesia; OH, onset hyperalgesia.

In the exploratory analysis of the combined data set from experiment 1 and 2 (Fig. 4), we found small but significant correlations between the offset and the onset effect with ±1°C stimulus range, *r*(60) = 0.27, *P* = 0.031 and between the offset and the onset effect with ±2°C stimulus range, *r*(60) = 0.27, *P* = 0.029.

## 4. Discussion

The aim of these 2 experiments was to examine if a hyperalgesic OH response could be induced by an inverted version of a well-documented hypoalgesic OA paradigm and to determine if different stimulus ranges affect the OA and OH responses. Here, OA was successfully elicited in OA_2°C_ in experiment 1, and in OA_1°C_ and OA_2°C_ in experiment 2. We also demonstrated that a larger stimulus range (ie, larger increase/decrease in temperature) produced larger OA effects. The latter result indicates a continuous stimulus-response relationship between stimulus range and the following reduction of pain. Grill and Coghill found a similar relationship in their first study of OA.^7^ It is reasonable to assume that if there is a temporal contrast enhancement mechanism for nociceptive input, it should be sensitive to the magnitude of the temporal contrast of the nociceptive input. Moreover, this result also indicates that a ±2°C design could be superior to a ±1°C design in studies where statistical power is an issue, for example, studies of clinical populations with small sample size and/or between-group comparisons. It is possible that a ±2°C design challenges the pain modulatory system more effectively and thereby has the potential to elucidate differences between patients with altered pain modulation and healthy controls.

Onset hyperalgesia was only induced in OH_2°C_ in experiment 1. Exploratory analysis suggests that the lack of significant results in experiment 2 could be the result of sex differences. Male participants in experiment 2 (there were only female participants in experiment 1) experienced less hyperalgesia during the OH_2°C_ compared with female participants. However, this finding should be interpreted carefully because of the exploratory nature of the analysis and the low sample size. Although these results are far from conclusive, it is possible that OH is a less stable phenomenon than OA. At first glance, this might seem surprising as one may think that a responsive pain system would yield significant results in both the hyperalgesic and hypoalgesic direction. Yet, we found that OA was more reliably induced than OH. The weak correlation between the OA and OH effects in the exploratory analysis further highlights the asymmetry between OA and OH. Although Alter et al. emphasized the similarities between OA and OH in their study, they did report measurements of the OA and OH effects that were only weakly correlated.^1^ Mørch et al.^9^ also found that decreases in noxious temperature lead to slower but larger changes in pain than increases in temperature, which led them to propose that there are different mechanisms underlying pain responses to increases and decreases of temperature. The weak correlation between OA and OH effects found in our study supports the notion of dual mechanisms.

A predictive coding perspective could also be useful in understanding the asymmetric response to rises and falls of noxious heat that we observed in the study. One important difference between the OH and OA conditions is that the latter involves temperatures 1°C or 2°C above T1 temperature (calibrated as 5 of 10 NRS). The brief but sharp rise in temperature in the OA conditions can be seen as a salient “learning signal” that affects pain modulation on return to T1 temperature. As the noxious input during the short increase of temperature deviates from the predicted sensation, an error signal may feed forward to adjust the perception and/or update the relevant generative models.^3^ Hence, the mismatch between top–down predictions of perceived pain and bottom–up noxious signals provides a mechanism for pain adaptation. In the case of OA, this adaptation is expressed as inhibitory modulation of noxious heat. However, in the case of OH, it is unclear if the brief decrease in temperature (−1°C or −2°C) may create a similarly salient surprise and motivate a subsequent adjustment of pain perception. This could also explain the discrepancy between our results and those of the previous study of OH. Although our OH design used a T1 temperature (during T1 and T3) calibrated to each participants' pain level of 5 NRS, Alter et al.^1^ used a 5 NRS +1°C as T1 temperature, which could result in a “saliency matched” learning signal for the OA and OH conditions, explaining the discrepancy between our and their finding.

A combined OA and OH protocol, which we use in this study, makes it possible to study different aspects of pain modulation by slightly modifying the stimulation sequence. For example, this combined protocol could be a useful tool to study the inhibitory/facilitatory balance in the pronociceptive and antinociceptive modulation profiles proposed by Yarnitsky, Granot, and Granovsky.^19^

In conclusion, the results from this study provide evidence for continuous stimulus-response relationship between the stimulus range (increase/decrease of temperature) and hypoalgesia related to the OA paradigm, and highlight the motivational role of the learning-signal in temporal contrast enhancement of pain. Future studies should determine if OA and OH represent dual mechanisms or if temporal contrast enhancement is symmetric.

## Disclosures

The authors have no conflicts of interest to declare.

Part of the results from the study has been presented as a poster and abstract on the EFIC conference in Valencia, 2019.
